# A chromosome-level genome assembly of the disco clam, *Ctenoides ales*

**DOI:** 10.1093/g3journal/jkae115

**Published:** 2024-05-28

**Authors:** Kyle E McElroy, Rick Masonbrink, Sivanandan Chudalayandi, Andrew J Severin, Jeanne M Serb

**Affiliations:** Department of Ecology, Evolutionary, and Organismal Biology, Iowa State University, Ames, IA 50011, USA; Genome Informatics Facility, Iowa State University, Ames, IA 50011, USA; Genome Informatics Facility, Iowa State University, Ames, IA 50011, USA; Genome Informatics Facility, Iowa State University, Ames, IA 50011, USA; Department of Ecology, Evolutionary, and Organismal Biology, Iowa State University, Ames, IA 50011, USA

**Keywords:** mollusca, bivalve, opsin, transposable element, mitochondria

## Abstract

The bivalve subclass Pteriomorphia, which includes the economically important scallops, oysters, mussels, and ark clams, exhibits extreme ecological, morphological, and behavioral diversity. Among this diversity are five morphologically distinct eye types, making Pteriomorphia an excellent setting to explore the molecular basis for the evolution of novel traits. Of pteriomorphian bivalves, Limida is the only order lacking genomic resources, greatly limiting the potential phylogenomic analyses related to eyes and phototransduction. Here, we present a limid genome assembly, the disco clam, *Ctenoides ales* (*C. ales*), which is characterized by invaginated eyes, exceptionally long tentacles, and a flashing light display. This genome assembly was constructed with PacBio long reads and Dovetail Omni-C^TM^ proximity-ligation sequencing. The final assembly is ∼2.3Gb and over 99% of the total length is contained in 18 pseudomolecule scaffolds. We annotated 41,064 protein coding genes and reported a BUSCO completeness of 91.9% for metazoa_obd10. Additionally, we report a complete and annotated mitochondrial genome, which also had been lacking from Limida. The ∼20Kb mitogenome has 12 protein coding genes, 22 tRNAs, 2 rRNA genes, and a 1,589 bp duplicated sequence containing the origin of replication. The *C. ales* nuclear genome size is substantially larger than other pteriomorphian genomes, mainly accounted for by transposable element sequences. We inventoried the genome for opsins, the signaling proteins that initiate phototransduction, and found that, unlike its closest eyed-relatives, the scallops, *C. ales* lacks duplication of the rhabdomeric G_q_-protein-coupled opsin that is typically used for invertebrate vision. In fact, *C. ales* has uncharacteristically few opsins relative to the other pteriomorphian families, all of which have unique expansions of xenopsins, a recently discovered opsin subfamily. This chromosome-level assembly, along with the mitogenome, is a valuable resource for comparative genomics and phylogenetics in bivalves and particularly for the understudied but charismatic limids.

## Introduction

The bivalve subclass Pteriomorphia includes many of the most economically important bivalves such as mussels, oysters, and scallops. Among the numerous morphological innovations in this clade are nonhomologous pallial eye types, including pigmented cups and compound eyes in Arcidae, cap eyespots in Ostreidae, mirror eyes in Pectinidae, and invaginated eyes in Limidae (Audino *et al*. 2020). The many origins of eyes in Pteriomorphia make this clade a compelling setting to study how novel traits arise and whether divergence in genetic architecture underlies this evolution.

To date, over 30 pteriomorphian genomes have been sequenced including at least one chromosome-level assembly from Arcida (Bai *et al*. 2019), Mytilida (Yang *et al*. 2021), Ostreida (Peng *et al*. 2020; Takeuchi *et al*. 2022), and Pectinida (Kenny *et al*. 2020). Outside of scallops (Pectinidae), no other eyed species have been sequenced, therefore limiting the possibility of comprehensive phylogenomic comparative studies on the evolution of eyes in this taxonomic lineage. Here, we present an annotated chromosome-level genome assembly of *Ctenoides ales* (*C. ales*) (Finlay, 1927), an eyed limid species. This genomic data will be valuable for exploring the repeated evolution of eyes in Pteriomorphia and represent the first genomic data from Limida, a diverse order of about 200 extant species (MolluscBase) commonly referred to as flame scallops, file clams, or file shells.

**Fig. 1.**
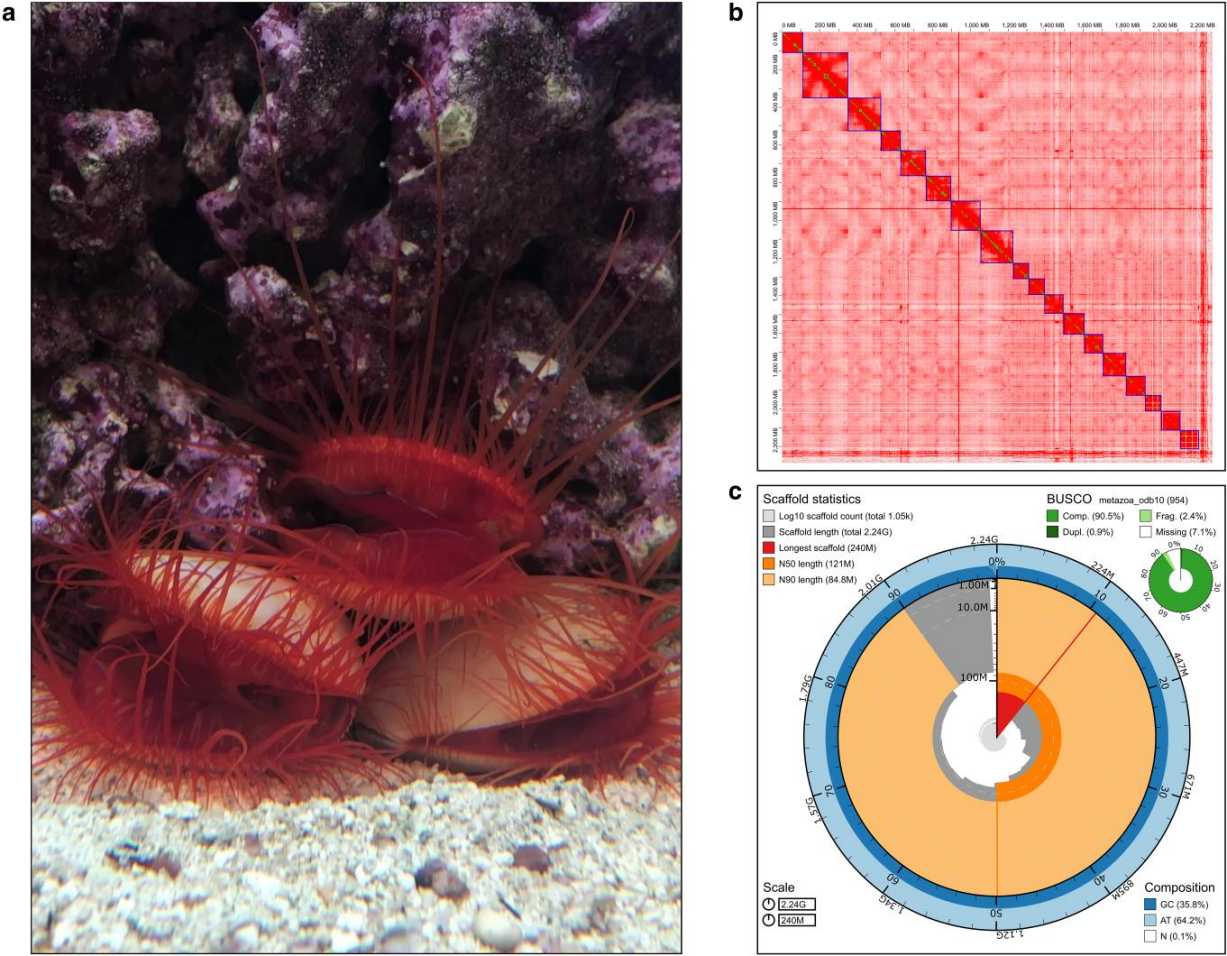
*Ctenoides ales* and summary of its genome assembly. a) Adult *Ctenoides ales* in aquarium setting, flashing light display visible in middle individual. Image credit: Jeanne M Serb. b) Hi-C contact map for *C. ales*, highlighting the 18 chromosomes recovered from the genome assembly. Darker shades (red) indicates higher density of contact, large (blue) and small (green) boxes denote chromosome and contigs, respectively. c) Snail plot summarizing key assembly statistics for final *C. ales* assembly with BUSCO results (−long –augustus parameters enabled).

The charismatic *C. ales* (Fig. 1a) is known as the “disco clam” or “electric flame scallop” for its flashing mantle display. Despite its bioluminescent-like appearance, this presumed antipredator display is actually the result of light reflecting from silica nanospheres incorporated into the mantle tissue (Dougherty *et al*. 2014). Limids are also known for their brightly colored mantle tissue lining the two valves, which is a source of chemical deterrent from predators (Dougherty *et al*. 2019). Long tentacles are used not only for sensory perception, but also for swimming and chemical defense (Mikkelsen and Bieler 2003; Donovan *et al*. 2004; Dougherty *et al*. 2019). At the base of these tentacles in many limid species are multiple “invaginated” eyes embedded in the mantle tissue (Bell and Mpitsos 1968; Mpitosos 1973; Morton 2000). Similar to scallops (Gorman and McReynolds 1969), limid eyes have two distinct retinas, a “proximal” and a “distal” retina that are made up of rhabdomeric and ciliary photoreceptor cells, respectively (Speiser *et al*. 2023), with opposing responses to light (Mpitosos 1973). However, the morphological characterization of limid eyes is complicated by differing interpretations and taxonomically narrow studies. Whether limids have spatial vision is still unknown (Speiser *et al*. 2023). Morphological and behavioral analyses suggest that *C. ales* has poor visual resolution and is unable to distinguish light directionality (Dougherty *et al*. 2017), in contrast to its closest eyed relatives, the scallops, equipped with image-forming eyes (Land 1965). Genomic comparisons across a variety of eyed and eyeless pteriomorphians should yield great insights into the molecular evolutionary process underlying the emergence of novel photoreceptive organs.

Gene duplication is an important source of new genetic information that can be used in the evolution of novel traits (Ohno 1970; Lynch and Conery 2000; Zhang 2003; Birchler and Yang 2022). Opsin duplication is important for the path to wider spectral sensitivity, particularly in vertebrates (e.g. Escobar-Camacho *et al*. 2020; reviewed in Hagen *et al*. 2023) and arthropods (Briscoe 2001; Briscoe *et al*. 2010; Bentley *et al*. 2016). Opsins are transmembrane G-protein-coupled receptor (GPCR) proteins that form photopigments by binding to a chromophore, typically a vitamin A-derived retinol, which upon light absorption starts a phototransduction cascade. Opsins are a phylogenetically diverse and widespread protein family with a variety of both ocular and extraocular light-dependent functions and light-independent functions (Terakita 2005; Shichida and Matsuyama 2009; Terakita and Nagata 2014; Moraes *et al*. 2021). Opsin classification is based on the specific G-protein an opsin activates (e.g. G_q_, G_t_, G_i_, G_o_, G_s_), the type of photoreceptor cell where it is expressed (e.g. ciliary vs rhabdomeric), and its phylogenetic placement (e.g. tetraopsins) (reviewed in Shichida and Matsuyama 2009; Porter *et al*. 2011). Although the phrase “visual opsin” has recently come into question (Feuda *et al*. 2022), typically vertebrates rely on ciliary (c-type) opsins that couple with G_t_ proteins for vision, bilaterian invertebrates use rhabdomeric (r-type) G_q_-opsins, and cnidarians use a distinct group of G_s_-opsins. Transcriptome sequencing has revealed multiple duplications of the rhabdomeric opsins (r-opsins) in scallops (Porath-Krause *et al*. 2016), which have a complex, image-forming mirror eye, raising the possibility that opsin duplication is characteristic of eyed pteriomorphian lineages. We explore this hypothesis by scanning the *C. ales* genome assembly for opsins and phylogenetically analyzing them in the broader context of Pteriomorphian opsin evolution.

## Materials and methods

### Sample collection and DNA extraction

Live *C. ales* individuals, acquired through the aquaria pet trade, were dissected from their shells, then whole animals were immediately flash frozen in liquid nitrogen and stored at −80C. Frozen adductor muscle tissue (400 mg) was homogenized into a fine power in liquid nitrogen and then DNA was extracted using a QIAGEN Genomic-tip protocol for tissue. Briefly, powdered tissue was lysed in QIAGEN G2 buffer at 50°C with RNAse A and Proteinase K for 2 hours, then the lysate was passed through a QIAGEN Genomic-tip by gravity. DNA was precipitated with 0.7 volumes of isopropanol, pelleted, and washed with 70% ethanol before resuspension.

### PacBio library and sequencing

DNA samples were quantified using Qubit 2.0 Fluorometer (Life Technologies, Carlsbad, CA, USA). The PacBio SMRTbell library (∼20 kb) for PacBio Sequel was constructed using SMRTbell Express Template Prep Kit 2.0 (PacBio, Menlo Park, CA, USA) using the manufacturer's recommended protocol. The library was bound to polymerase using the Sequel II Binding Kit 2.0 (PacBio). Sequencing was performed on PacBio Sequel II 8 M SMRT cells.

### Dovetail Omni-C library preparation and sequencing

Frozen adductor muscle tissue (50 mg) from the same individual used for PacBio sequencing was pulverized to a powder in liquid nitrogen then used as input to prepare the Dovetail Omni-C library. Chromatin was fixed in place with formaldehyde in the nucleus and then extracted. Fixed chromatin was digested with DNAse I, and chromatin ends were repaired and ligated to a biotinylated bridge adapter followed by proximity ligation of adapter containing ends. After proximity ligation, crosslinks were reversed, and the DNA purified. Purified DNA was treated to remove biotin that was not internal to ligated fragments. Sequencing libraries were generated using NEBNext Ultra enzymes and Illumina-compatible adapters. Biotin-containing fragments were isolated using streptavidin beads before PCR enrichment of each library. The library was sequenced on an Illumina HiSeqX platform to produce approximately 30x sequence coverage. Then HiRise used MQ > 50 reads for scaffolding.

### RNA isolation and sequencing

Flash frozen whole-animal tissue was stored at −80C until preparation for RNA isolation. For every animal used, a sample of adductor, mantle, and eyes was prepared. Isolated tissue was first ground into a homogenous powder using an OPS Diagnostics LLC CryoGrinder^TM^ System and then stored at −80°C until RNA isolation. Total RNA was isolated from ground tissue powder using an E.Z.N.A Total RNA Kit II (Omega BIO-TEK). RNA purity was assessed with a Nanodrop ND-1000 Spectrophotometer and ND-1000 3.2.1 software. RNA integrity and quantity were determined with an Agilent 2100 Bioanalyzer (Agilent Technologies). Libraries for sequencing were prepared with the NEBNext Ultra Directional RNA Library Prep Kit (Illumina), following poly(A) mRNA enrichment with Oligo dT Beads, and then sequenced (2 × 150 bp reads) on an Illumina NovaSeq 6000 at Iowa State University's DNA Facility.

### Draft assembly

Approximately 290.8 Gb of PacBio Continuous Long Reads (CLR) reads were used as an input for assembly by WTDBG2 v2.5 (Ruan and Li 2020) with genome size set to 1.7 Gb, minimum read length 20,000, and minimum alignment length 8,192. Additionally, realignment was enabled with the -R option and read type was set with the option -x sq. Blast results of the WTDBG2 output assembly against the NCBI NT database were used as input for Blobtools v1.1.1 (Laetsch and Blaxter 2017) and scaffolds identified as possible contamination were removed from the assembly. Finally, purge_dups v1.2.3 (Guan *et al*. 2020) was used to remove haplotigs and contig overlaps.

### HiRise scaffolding

The de novo WTDBG2 assembly and Dovetail Omni-C library reads were used as input data for the proximity ligation-based genome-scaffolding pipeline, HiRise (Putnam *et al*. 2016). Dovetail Omni-C library sequences were aligned to the draft input assembly using (https://github.com/lh3/bwa) (Li 2013). The separations of Dovetail Omni-C read pairs mapped within draft scaffolds were analyzed by HiRise to produce a likelihood model for genomic distance between read pairs, and the model was used to identify and break putative misjoins, to score prospective joins, and make joins above a threshold.

### Final assembly

Dovetail assembly scaffolds were scaffolded using Juicer v1.5.7 (Durand *et al*. 2016), 3D-DNA v180114 (Dudchenko *et al*. 2017), Juicebox v1.11.08 (Robinson *et al*. 2018), based on BWA 0.7.17 alignments of Omni-C reads. Contigs that could not be placed in pseudomolecules were subjected to redundancy filtering using coordinates from two criteria: mapping contigs to the pseudomolecules with Minimap v2.2 and repeats identified as described in the following. These coordinates were merged using Bedtools (Quinlan and Hall 2010) merge, and when a contig could achieve 90% identity across 90% of the contig length to a pseudomolecule, it was removed. Additionally, unplaced contigs were again assessed as potential contamination with Blobtools v2.2.0, based on blastn v2.11.0 (Camacho *et al*. 2009) hits against the NCBI nt database (downloaded May 30, 2020) and mapping coverage from Minimap v2.2 (Li 2018) alignments of PacBio subreads. A final assessment of genome completeness following contig elimination was measured with BUSCO v5.1.2 (Waterhouse *et al*. 2018) using metazoa_odb10, mollusca_odb10, and eukaryota_odb10.

### Transposable element characterization

De novo repeat identification was conducted with RepeatModeler v2.0.2 (Flynn *et al*. 2020), utilizing RepeatScout v1.0.6 (Price *et al*. 2005), RECON v1.08 (Bao and Eddy 2002), LTR_Retriever v2.9.0 (Ou and Jiang 2018), and the Extensive de novo TE Annotator (EDTA) v2.0.1 (Ou *et al*. 2019), which uses three pipelines for TE discovery based on structural characteristics of terminal inverted repeated (TIR) DNA transposons, long terminal repeat (LTR) retrotransposon, and helitrons. Next, TEsorter v1.4.6 (Zhang *et al*. 2022) was used to classify sequences from the RepeatModeler and EDTA output based on REXdb v. 3 HMM profiles and CD-HIT-EST v4.8.1 was used to combine classified sequences and reduce redundancy between the two sets of repeats (Li and Godzik 2006; Fu *et al*. 2012). Because SINEs lack any coding sequences and no structural detection pipeline is included in EDTA, the sequences annotated as SINEs by RepeatModeler were queried against Repbase online (Bao *et al*. 2015) with CENSOR and kept sequences matching SINEs with scores >250. We combined the TEsorter classified sequences with SINEs and the remaining EDTA sequences identified via structural features (e.g. LTRs) into a single TE library for each species, and then masked each genome with its specific TE library using RepeatMasker v4.1.2-*P* (Smit *et al*. 2015). We used the RepeatMasker script buildSummary.pl to summarize the TE content of each genome and calcDivergenceFromAlign.pl to collect the Kimura substitution levels of TE copies to generate a repeat landscape. For comparison, we performed these analyses on the *C. ales* genome and other available pteriomorphian genome assemblies (see what follows).

### Gene prediction and functional annotation

Gene annotation was performed with BRAKER v2.1.2 (Brůna *et al*. 2021) using AUGUSTUS v3.3.2 (Stanke and Waack 2003; Stanke *et al*. 2006) and GeneMark v4.38 (Brůna *et al*. 2020). First, AUGUSTUS was trained via BUSCO v5.1.2 (−long –augustus –auto-lineage-euk) run on the *C. ales* genome assembly. Then, RNA-seq reads were mapped to a softmasked *C. ales* genome assembly with STAR v2.5.3a (Dobin *et al*. 2013) to generate splice-aware alignments for GeneMark-ET training in the BRAKER2 pipeline. The resulting gene annotations were further refined using Mikado v2.3.2 (Venturini *et al*. 2018) with Transdecoder (Haas *et al*. 2013) and BLAST + v2.11.0. Briefly, high-quality splice-junctions from the RNA-seq mapping results identified by Porticullis v1.2.2 (Mapleson *et al*. 2018) and protein alignments based on 283,363 Bivalvia proteins downloaded from Uniprot and mapped to the *C. ales* genome with Genomethreader v1.7.3 (Gremme *et al*. 2005) were used to identify the best transcript models with Mikado. Functional gene annotations were created via Diamond v2.0.4 (Buchfink *et al*. 2015) searches to NCBI NR (downloaded May 3, 2021), Uniprot/Swissprot (downloaded May 28, 2022), and Interproscan v5.38 (Jones *et al*. 2014).

### Phylogenetic analysis

Protein sequences were collected from annotated bivalve genomes, including 11 other pteriomorphian species and 2 outgroups (*Mercenaria mercenaria* and *Sinonovacula constricta*) to phylogenetically compare the protein coding content of the *Ctenoides ales* genome (Supplementary Table 1). A total of 508,333 proteins were analyzed with OrthoFinder v2.5.4 (Emms and Kelly 2019). From these results, 1,156 proteins were identified as single-copy and present in all 14 species analyzed. We used these protein sequences to produce a species tree. The amino acid sequences identified as single-copy and complete for all 14 species were aligned using mafft v7.481 (−auto) (Katoh *et al*. 2002; Katoh and Standley 2013). Next, we trimmed the alignments with trimal v1.4.rev15 (-automated1) (Capella-Gutiérrez *et al*. 2009). Then we used IQtree2 v2.1.3 (Nguyen *et al*. 2015; Minh *et al*. 2020) with modelfinder (-m MFP) (Kalyaanamoorthy *et al*. 2017) to generate maximum-likelihood trees for each trimmed protein alignment and a summary file of protein substitution models used. The substitution model results were combined with a gene partition file generated with catsequences (V1.3; 10.5281/zenodo.4409153) and used as input for a partitioned IQtree2 ML analysis (Chernomor *et al*. 2016). Branch support was evaluated by ultrafast bootstrap (Hoang *et al*. 2018), SH approximate likelihood ratio test, and approximate Bayes test features in IQtree2 (-B 1000 –alrt 1000 –abayes) (Anisimova *et al*. 2011).

A second species tree was generated to include multiple taxa per pteriomorphian family where high-quality genomes were available but not genome annotations (i.e. additional Arcidae). This was done to account for variation within taxonomic families in downstream characterization of transposable element and opsin content. As in McElroy *et al*. (2023), BUSCO was used to predict conserved protein sequences to construct a species tree independent of genome annotations. We downloaded genome assemblies from 12 pteriomorphian species, three representatives from the families Arcidae, Pectinidae, Ostreidae, and Mytilidae, along with four other bivalve species as outgroups (accessions and assembly statistics listed in Supplementary Table 2). We ran BUSCO v5.2.2 on each of these 16 genome assemblies using the metazoa_odb10 database (BUSCO scores listed in Supplementary Fig. 1, Supplementary Table 2). We then used the 177 BUSCO amino acid sequences identified as single-copy and complete for all 16 species as input for a maximum-likelihood analysis following the same methods described for the first species tree.

### Synteny between *C. ales* and *P. maximus*

Synteny was determined using i-ADHoRe v3.0.01 (Proost *et al*. 2012) using the OrthoFinder2 results and their respective assemblies and annotations. Synteny was only performed for scaffolds larger than 1 Mb. The following parameters were included in the i-ADHoRe config: blast_table = black.blastTable, prob_cutoff = 0.001, anchor_points = 3, number_of_threads = 36, visualizeAlignment = false, output_path = out_5, alignment_method = nw, gap_size = 25, cluster_gap = 50, level_2_only = true, and q_value = 0.9. The function dashbio.Circos in the dashbio library was used to visualize the synteny in a circos plot.

### Opsin identification and analysis

We identified opsins via the Phylogenetically Informed Annotation tool (PIA; Speiser *et al*. 2014) from the *C. ales* gene models and the homology-based de novo gene prediction with the BITACORA pipeline v1.3 (Vizueta *et al*. 2020) using GeMoMa (Keilwagen *et al*. 2016, 2018) and a database of molluscan opsin sequences described in McElroy *et al*. (2023). For PIA, we used the modified version from https://github.com/MartinGuehmann/PIA2 and the Light Interacting Toolkit (LIT_1.1; opsins using r_opsin_20_rtrans.fas for opsin classification). For further opsins classification and phylogenetic comparison, we included McElroy *et al*. (2023) opsins for the following species: *Argopecten irradians*, *Pecten maximus*, *Mizuhopecten* (*Patinopecten*) *yessoensis*, *Scapharca broughtinii*, *Scapharca kagoshimensis*, *Tegillarca granosa*, *Perna viridis*, *Mytilus coruscus*, *Mytilus galloprovincialis*, *Crassostrea gigas*, and *Crassostrea virginica*. We additionally generated opsin models for *Ostrea edulis*. For all opsins, we manually inspected the sequences to ensure high quality (i.e. intact GPCR Class A 7tm_1 domain and containing the K296 position). For outgroup sequences, we used melatonin receptors and the opsin-like sequences from Placozoa, “placopsins” (Feuda *et al*. 2012), along with the more recently described GPRC relative of opsins found in lophotrochozoans, the “pseudopsins” (De Vivo *et al*. 2023). We aligned the opsin and outgroup amino acid sequence for the 13 pteriomorphian species using mafft [–maxiterate 1000 –genafpair]. We then generated a maximum-likelihood tree in IQtree2 with modelfinder (best-fit model Q.yeast + F + R8 according to Bayesian Information Criterion) that was evaluated with ultrafast bootstrap, SH approximate likelihood ratio test, and approximate Bayes test features in IQtree2 (-B 1000 –alrt 1000 –abayes).

### Mitogenome assembly and assembly

To characterize the mitochondrial genome, a partial *C. ales* cytochrome c oxidase subunit I (COI (MF540379.1) was queried against the final genome assembly using BLASTn. This search yielded no returns. Next, the draft, unfiltered WTDBG2 assembly was searched, returning a single contig approximately 18 kb long containing the partial COI sequence. The PacBio reads were mapped to the mitochondrial contig with minimap2 v2.14-r883 (Li 2018). The aligned reads were then extracted and assembled with Flye v2.9 (Kolmogorov *et al*. 2019), which generated a single circular contig 20,859 bp long. This contig was then annotated on the MITOS [Genetic Code 5: Invertebrate Mitochondrial (Bernt *et al*. 2013)] and MITOS2 (Donath *et al*. 2019) web servers (RefSeq 89 Metazoa; Genetic Code 5: Invertebrate Mitochondrial). Additionally, the mitochondria genome was scanned for open reading frames (ORFS) with OFRfinder on NCBI [https://www.ncbi.nlm.nih.gov/orffinder/ (Genetic Code 5: Invertebrate Mitochondrial; “ATG” and alternative start codons)]. The ORFfinder results were used to characterize the complete coding sequences for each of the 12 proteins identified. ARWEN v1.2 (Laslett and Canbäck 2008) was additionally used to evaluate tRNAs predicted by MITOS and potentially identify the 2 tRNAs classified as “missing” by MITOS annotation. The PacBio and forward reads from the Omni-C Illumina sequences were mapped [minimap2 and bwa-mem2 (Vasimuddin et al. 2019), respectively] to evaluate the apparent duplication of the OH sequence. To visualize the duplication by coverage of the short-read mapping, a version of the mitogenome was generated with 1 copy of the duplicated region hard masked to prevent reads from aligning to both duplication regions.

## Results and discussion

### Genome assembly and completeness analysis

Dovetail performed a de novo genome assembly of *Ctenoides ales*. Approximately 290 billion bases of PacBio CLR were used in the assembly (14,188,342 reads, average length = 20.5Kb). The WTDBG2 assembler generated a draft assembly, which was subsequently scaffolded with 602 million Omni-C reads (Supplementary Table 3 for intermediate stats). These reads were then used to further scaffold the Dovetail genome using Juicer, 3D-DNA, and manually corrected using Juicebox. After eliminating haplotigs and contaminants, the final assembly contained 1,049 scaffolds and 2.2 billion bases. Among these scaffolds, the 18 largest correspond to chromosomes, accounting for 99.16% of the total nucleotide content (Fig. 1b). The assembly's scaffold N50 value is 121 Mb, with the longest scaffold measuring 240 Mb (Fig. 1c). The completeness of this assembly is exemplified by the 98.56% mapping rate of the PacBio long reads against it, which amounts to approximately 100x coverage. Furthermore, the assembly exhibits a BUSCO completeness score of 91.9% using the metazoa_odb10 gene set (Fig. 1c) and an 85.4% score based on the mollusca_odb10 gene set. This BUSCO-based assembly quality is in line with other pteriomorphian assemblies (Supplementary Fig. 1), including scallops; however, the genome is considerably larger. For example, the *Pecten maximus* genome released in 2019 by the Wellcome Sanger Institute is chromosomal with 19 chromosomes and 3,983 scaffolds containing 918 million bases (Kenny *et al*. 2020), making our *C. ales* genome assembly over two times larger (see Supplementary Table 2 for assembly size comparisons). We further examined the genome size disparity between *C. ales* and other pteriomorphians in the context of gene content, genome duplication, and transposable elements.

### Gene content and phylogenetic analysis

RNA-seq data were generated at the Iowa State University DNA Facility using eye, mantle, and adductor muscle tissues from 10 *C. ales* specimens and used to annotate genes with BRAKER2, which resulted in 43,799 genes. Further refinement of these gene models with Mikado resulted in 41,064 gene models with an average gene length of 23,950 bp (see Table 1 for further details). We ran BUSCO in protein mode on our annotation results, yielding high completeness scores for metazoan_odb10 (C:89.2%, F:6.0%, M:4.8%, n:954) and mollusca_odb10 (C:81.5%, F:4.6%, M:13.9%, n:5295) that were comparable to the scores for the genome assembly. We aligned amino acid sequences from predicted genes against the NR and Swissprot databases and assigned functional information to 77.7% of the genes. The metazoan BUSCO scores of the gene annotation indicate a high degree of completeness at 89.2%, which is qualitatively similar to the results from the genome assembly. These gene model predictions suggest that *C. ales* may have nearly 50% more genes than is typical for some pteriomorphians. The first scallop genomes, *Mizuhopecten* (*Patinopecten*) *yessoensis* and *Chlamys farreri,* were described as having 26,415 (Wang *et al*. 2017) and 28,602 (Li *et al*. 2017), respectively. While the scallop *Pecten maximus* was initially reported to have 67,741 protein coding genes (Kenny *et al*. 2020), Zeng *et al*. (2021) identified 26,995 genes. Arcidae gene reports tend to have similar numbers of genes as scallops, e.g. 24,045 in *Scapharca broughtonii* (Bai *et al*. 2019) and 24,398 in *Tegillarca granosa* (Bao *et al*. 2021). The number of protein coding genes predicted in *C. ales* is more comparable with genome annotations from Mytilidae and Ostreidae genomes, which tend to contain between 30,000 and 40,000 genes (e.g. Peñaloza *et al*. 2021; Yang *et al*. 2021). Notably, transposable elements (TEs) do not appear to make up a substantial portion of the predicted genes as only 1,825 genes (∼4% of total) have annotation terms from likely TEs (“transposase,” “reverse transcriptase,” “helicase,” “LINE,” “integrase,” and “RNAse H”). Therefore, the relatively high number of genes predicted in this genome are not likely inflated by TEs annotated as genes. Variation in gene model prediction strategies may account for some of the differences in gene totals, as fragmentation of lowly expressed genes may have occurred. Additionally, factors such as genome size and evolutionary processes may also contribute to these differences.

**Table 1.** Summary statistics of *Ctenoides ales* genome and annotation.

Assembly stats		Annotation stats	
Number of scaffolds	1,049	Number of genes	41,064
Length of assembly	2,237,305,496 bp	Number of mRNA	44,726
Longest scaffold	240,195,547 bp	Mean gene length	23,950 bp
Scaffold L50/N50	7/121,434,358 bp	Mean CDS length	1,346 bp
Scaffold L90/N90	16/84,817,633 bp	Mean exon per gene	7.2
Scaffold CG Content	35.77%	Mean introns per gene	6.2
Scaffold N content	0.09%	Mean exon length	188 bp
Number of contigs	8,191	Mean intron length	4,375 bp
Longest contig	7,630,288 bp	Total CDS length	50,641,044 bp
**Contig L50/N50**	508/1,207,478 bp		
**Contig L90/N90**	2476/133,914 bp		

**Fig. 2.**
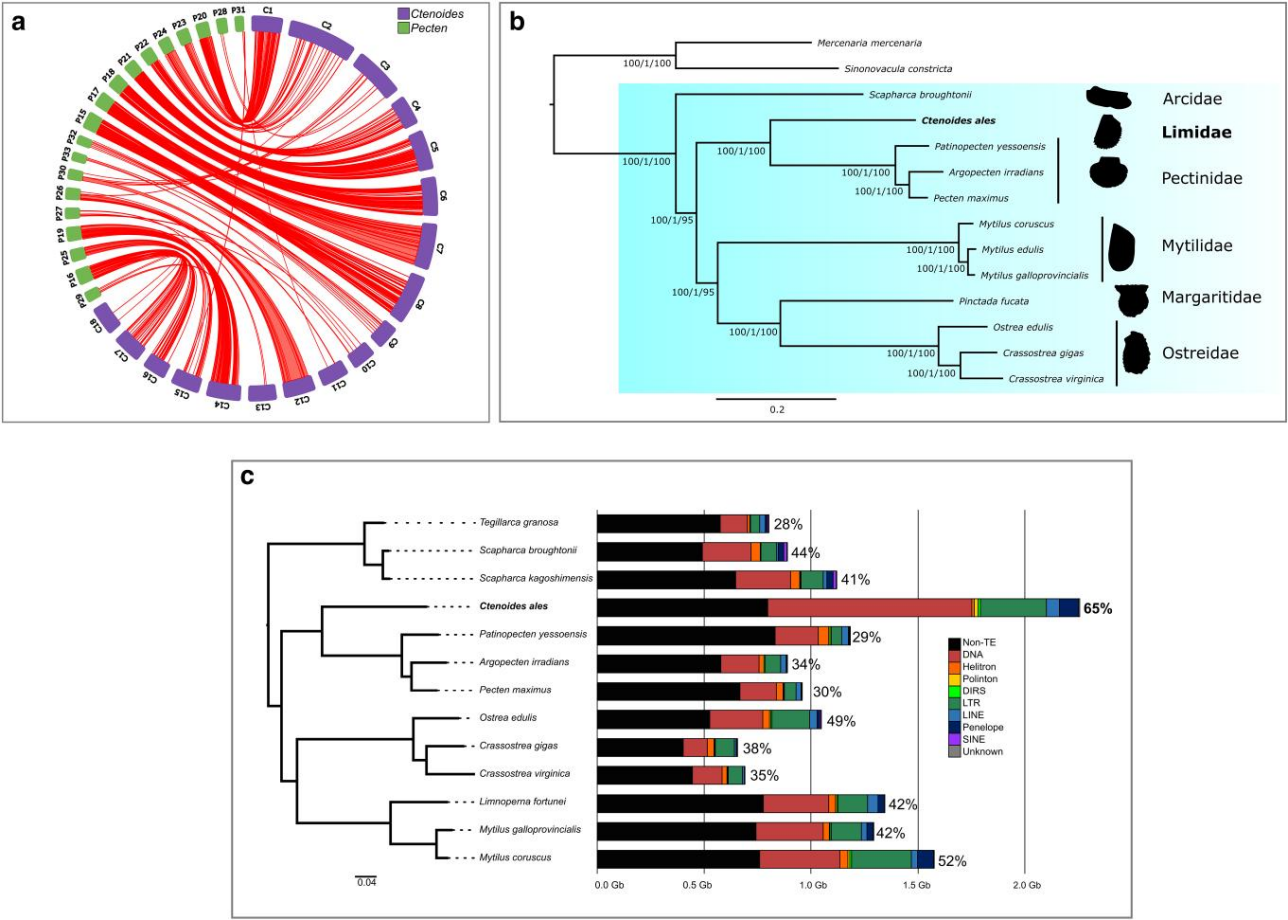
Comparative genomics with other bivalves. a) Circos plot of syntenic regions (red arcs) between *C. ales* (in purple) and *P. maximus* (in green). Scaffold names were shortened to their unique numbering and the name portion replaced with a C for *Ctenoides* or a *P* for *Pecten*. b) Phylogenetic placement of *Ctenoides ales* among bivalves. Maximum-likelihood species tree generated with IQtree2 based on a partitioned amino acid supermatrix from 1,156 single-copy orthologs identified from OrthoFinder2 recovered from all 14 species displayed. Branch values are SH-aLRT % support (with 1,000 replicates)/aBayes probability/UFBoot support % (with 1,000 replicates). c). Genome assemblies for 13 pteriomorphian species characterized by TE amount by distinct types of TEs and “non-TE” portion of the genome in black; % given per species is the genomic proportion of TEs.

To phylogenetically compare the protein coding content of the *Ctenoides ales* genome, we collected protein sequences from annotated bivalve genomes, including 11 pteriomorphians and 2 outgroup species (*Mercenaria mercenaria* and *Sinonovacula constricta*). We analyzed a total of 508,333 proteins with OrthoFinder2 (Emms and Kelly 2015; Emms and Kelly 2019), which resulted in clustering 91.5% of the proteins into 465,163 orthogroups (summarized in Supplementary Table 4). Only 7.4% of the proteins belong to species-specific orthogroups. For *C. ales*, 33,033 of the 39,079 protein sequences analyzed were placed into orthogroups, 5,230 of which were specific orthogroups to *C. ales*, meaning that the majority (71.1%) of protein-coding genes from our annotation are shared with other species (e.g. Supplementary Fig. 2). A lack of close relatives may have influenced these results, as species with the highest proportion of genes in species-specific orthogroups are those that are the sole representatives of a family (*C. ales*: 13.4%, *M. mercenaria*: 19.5%, and *P. fuctata*: 16.3%). We used the 1,156 single-copy orthologs found in all 14 species to reconstruct the species phylogeny using maximum-likelihood analysis with IQTREE2. This phylogeny had high support values at all nodes and met expectations of species relationships, including *C. ales* representing Limidae as a sister lineage to Pectinidae (e.g. Audino *et al*. 2020) (Fig. 2b). Together, these analyses of the *C. ales* gene content are indicative of a high-quality annotation for this first limid genome. Furthermore, although the gene count for *C. ales* is relatively high within Pteriomorphia, protein coding sequence accounts for 50Mb, or 2.3%, of the total genome assembly length, therefore contributing very little to its large genome size (e.g. the scallops *P. maximus* and *Mi. yessoensis* have about 41Mb of coding sequence, each). More genome assemblies from limid species will be needed to clarify if the *Ctenoides* genome is broadly representative of species in Limida for gene content and determine whether gene expansions in this lineage predated, accompanied, or followed its genome size increase.

### Synteny shows no whole-genome duplication events

To investigate the potential involvement of whole-genome duplication (WGD) in the larger genome size of *Ctenoides ales* than scallops and other pteriomorphian species, we conducted a synteny comparison with the king scallop, *Pecten maximus*. The expectation was to observe a majority of chromosomes exhibiting synteny from one chromosome in *P. maximus* to two chromosomes in *C. ales*, indicating a WGD event. We found no evidence of WGD. Instead, the results revealed a one-to-one correspondence for 12 chromosome pairs (*Ctenoides (C)*:*Pecten (P)*): C2:P24, C3:P30, C4:P22, C5:P21, C6:P18, C7:P17, C8:P15, C9:P32, C12:P26, C14:P19, C15:P25, and C18:P29 (Fig. 2a).

Considering *C. ales* possessing one less chromosome, a fusion event was anticipated. The circos plot demonstrated C1 mapping to P20 and P23, but an inverse pattern was also identified where C15 and C17 both mapped to P16 (Fig. 2a). This observation highlights the occurrence of genomic rearrangement since the divergence of these species from their common ancestor. Genomic rearrangement is further supported by the diminished or fragmented synteny observed between chromosomes C10, C11, and C13 in *C. ales* and P27, P28, P31, and P33 in *P. maximus* (Fig. 2a). The circos plot's syntenic relationships reveal a high degree of synteny between the two species, accompanied by some genomic rearrangement. However, there was no evidence of a WGD event to account for the substantially larger genomic content in *C. ales*.

In general, high degrees of synteny have been observed across much of Pteriomorphia, including between scallop species (Han *et al*. 2022), between scallop and ark clam genomes (Bao *et al*. 2021), and scallop vs Mytilidae comparisons (Yang *et al*. 2021). The most extensive genome rearrangements in Pteriomorphia are in the oysters, which have notably fewer chromosomes (e.g. Gundappa *et al*. 2022) than groups such as scallops. Genomic evidence of past WGD has been reported from pteriomorphian genomes (Corrochano-Fraile *et al*. 2022), but these duplication events have not been placed phylogenetically, making it unclear whether these apparent WGD are shared across Pteriomorphia or occurred in parallel among separate lineages. Our analysis of the first limid genome indicates that no genome duplication has occurred in either Pectinidae or Limidae since their split. Furthermore, while the chromosomes of *C. ales* are much larger than *P. maximus*, gene order has largely been preserved between these lineages.

### Transposable elements contribute to large genome size in *Ctenoides*

The *C. ales* genome is twice as large as some genomes from Pectinidae (Fig. 2a), despite no evidence of WGD. To determine if the size difference was due to repetitive elements, we characterized repeat content from *C. ales* and 12 other pteriomorphians (Supplementary Table 5) with RepeatModeler, EDTA, and RepeatMasker. We found that much of the difference in genome size across Pteriomorphia is attributable to transposable elements. Based on our estimation, about 65% of the *C. ales* genome is made up of TEs, with DNA transposons making up 40% of the genome (summarized in Table 2). The TE content of *C. ales* is about twice that of the Pectinidae, and greater than any of the 12 other pteriomorphian genomes analyzed here (Fig. 2a). The repeat landscape of the *C. ales* genome reveals a large, ancient burst of TE activity, mainly in DNA transposons and a more recent burst in LTR *Gypsy* elements (Fig. 2b). We also observed far more “intact” (i.e. full structural and coding components) long terminal repeat (LTR) elements from the EDTA analysis than the other species analyzed, which also reflects recent activity of these TEs, such that they have not degraded or been excised from the genome (Supplementary Table 6).

**Table 2.** Transposable element content in *Ctenoides ales* genome.

Class	Family	Count	bp	Genome %
**TIR**	**Total**	**4,918,972**	**920,785,650**	**41**.**2**
	CACTA	1,271,100	182,680,333	8.2
	Merlin	601	277,138	0.0
	MuDR_Mutator	2,187,690	440,892,520	19.7
	*P*	367	56,747	0.0
	PIF_Harbinger	261,453	34,285,779	1.5
	PiggyBac	638	284,541	0.0
	Sola2	223	93,940	0.0
	Tc1_Mariner	464,116	121,158,874	5.4
	hAT	732,784	141,055,778	6.3
**MITE**	**Total**	**289,357**	**35,456,827**	**1**.**6**
	CACTA	2,4778	3,052,430	0.1
	MuDR_Mutator	21,2367	23,590,962	1.1
	PIF_Harbinger	8,601	713,658	0.0
	Tc1_Mariner	2,775	218,873	0.0
	hAT	4,0836	7,880,904	0.4
**DNA (TIR & MITE)**	**Total**	**5,208,329**	**956,242,477**	**42**.**7**
**Helitron**		79,069	10,440,877	0.5
**Maverick**		36,882	18,385,871	0.8
**DIRS**		17,555	10,406,517	0.5
**LTR**	**Total**	**1,174,081**	**309,813,718**	**13**.**8**
	Bel-Pao	65,336	19,418,066	**0**.**9**
	Copia	119,994	17,947,770	**0**.**8**
	Gypsy	297,782	132,963,481	**5**.**9**
	unknown	690,969	139,484,401	**6**.**2**
**LINE**		**129,954**	**59,550,410**	**2**.**7**
**Penelope**		**247,032**	**90,026,897**	**4**.**0**
**SINE**	5S	**622**	**87,775**	**0**.**0**
**unknown**		**20,928**	**4,675,207**	**0**.**2**
**Total TEs**		**6,780,946**	**1,420,396,484**	**65**.**2**

TIR, terminal inverted repeat; MITE, miniature inverted-repeat transposable element; DIRS, Dictyostelium Intermediate Repeat Sequence; LINE, long interspersed nuclear element; SINE, short interspersed nuclear element.

Several classes of TEs are far more abundant in *C. ales* than other pteriomorphians, particularly DNA transposons and LTR retrotransposons, and, to a lesser degree, LINEs and Penelope retrotransposons. Helitrons are less abundant in *C. ales* than other species analyzed here (Supplementary Table 5). SINEs appear to have had very little activity outside of Arcida (Fig. 2d, Supplementary Fig. 3), including *C. ales*. Based on the repeat landscapes, there has likely been greater TE activity more recently in the *C. ales* genome than *P. maximus* (Supplementary Fig. 3). We generally observed similar TE content within families, e.g. in Pectinidae *A. irradians*, *P. maximus*, and *Mi. yessoensis* differed by 5% across the three species. Within-family differences in TE content still typically varied less between species in the same genus, e.g. *Crassostrea*, but *M. galloprovincialis* appears to have more TEs than *M. coruscus* (Fig. 2a). With *C. ales* being the first limid genome sequenced, it will be important to sequence more broadly from Limida to have a thorough account of how and when this genomic expansion occurred.

TEs are important drivers of genome size evolution (Kidwell 2002) and can account for drastic variation in genome size across closely related species (e.g. Naville *et al*. 2019; Wong *et al*. 2019), but the factors influencing differential accumulation and loss of TEs across taxa are still an ongoing area of research. Population genetic theory (Lynch 2007) and empirical data (e.g. Szitenberg *et al*. 2016) point toward genetic drift as a powerful force influencing TE accumulation. Therefore, changes in effective population size could explain differences in TE content across taxa. Other factors, such as decreased TE silencing (Liu *et al*. 2022), may also contribute to TE expansion. The genome expansion we found in *C. ales* along with recent evidence for highly variable TE content across bivalves (Martelossi *et al*. 2023) highlight the importance of this genomically understudied group of animals for exploring the genomic and biological influences on TE evolution.

### Relatively few opsins in the *C. ales* genome

**Fig. 3.**
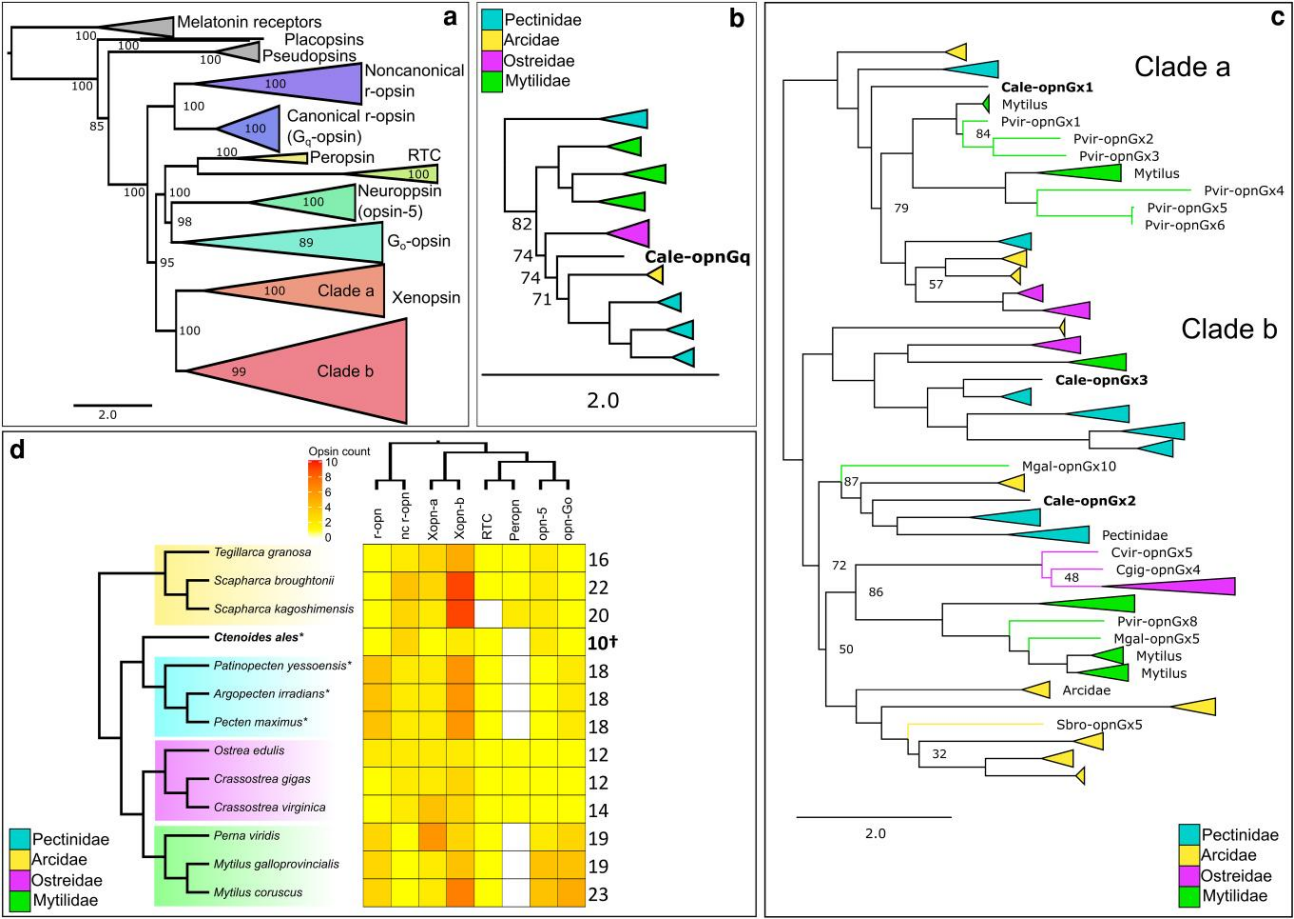
Summary of opsin content in *Ctenoides ales* and other pteriomorphian bivalves. a) ML phylogenetic tree of opsins from 13 pteriomorphian genomes, including *C. ales*. Ultrafast bootstrap (UFboot) support is shown (Supplementary Fig. 4). Subtrees of b) “canonical” G_q_-coupled r-opsins (“opnGq”), and c) xenopsins (“opnGx”). In b) and c), UFBoot support < 90 displayed; branches collapsed and color coded according to shared opsin duplicates within families; *C. ales* opsins noted in bold as “Cale-opn”. d) Heatmap reflects number of genes from each of 8 opsin groups (r-opn: rhabdomeric G_q_-coupled opsin; nc r-opn: noncanonical r-opsin; Xopn-a/b: xenopsin clades a and b; RTC: retinochrome; Peropn: peropsin; opn-5: neuropsin; opn-Go: G_o_-coupled opsin). Phylogenetic relationship among opsin groups based on Fig. 3a. Total numbers of opsins per species reported right of heatmap. Shading around taxa according to family color coding in previous panels. *eyed species. †*C. ales* count includes the partial noncanonical r-opsin and G_o_-opsin.

Using the Phylogenetically Informed Analysis tool with the light-detection toolkit (Speiser *et al*. 2014), we identified 6 opsins from our genome-wide annotation (1 of each: canonical r-opsin, noncanonical r-opsin, xenopsin, G_o_-opsin, retinochrome, and neuropsin) and an additional 2 complete xenopsins from the BITACORA pipeline output. We also found 2 partial opsin sequences (a noncanonical r-opsin and a G_o_-coupled opsin) from the BITACORA output containing the K296 residue but a truncated seven-transmembrane protein domain. To add evolutionary context for these *C. ales* opsins, we generated an ML phylogenetic tree that included opsins from 12 other pteriomorphian species (Fig. 3a, Supplementary Fig. 4). As outgroup proteins in this analysis, we included melatonin receptors and the placozoan opsin-like sequences “placopsins” (Feuda *et al*. 2012), along with the recently described group of closely related GPCRs in lophotrochozoans, “pseudopsins” (De Vivo *et al*. 2023).

The opsins in the *C. ales* genome belong to the major opsin groups present in mollusks: r-opsin (canonical and noncanonical), xenopsin, neuropsin, G_o_-opsin, and retinochrome (Ramirez *et al*. 2016) (Fig. 3d). There does not appear to be a peropsin (Fig. 3d) in the *C. ales* genome, which is also absent in Pectinidae and Mytilidae, but present in Ostreidae and Arcidae. The absence of perops in *C. ales* is likely a shared loss with Pectinidae. Compared with other species in Pteriomorphia, the limid, *C. ales* has a small opsin repertoire (Fig. 3d). Unlike other families in Pteriomorphia, this representative limid lacks any lineage-specific opsin duplications (Fig. 3d), even considering the partial noncanonical r-opsin and G_o_-opsin (Supplementary Fig. 4). The xenopsin group is particularly expansion prone in Pteriomorphia, as an independent series of lineage-specific duplications are present in genomes from Pectinidae, Arcidae, Mytilidae, and Ostreidae (McElroy *et al*. 2023). However, the three *C. ales* xenopsins are each phylogenetically located in separate clades of this opsin subfamily (Fig. 3c), making lineage-specific paralogous duplication highly unlikely. Very little is known about xenopsins, as they were only recently recognized as a distinct group of opsins sister to the cnidarian “cnidopsins” (Ramirez *et al*. 2016). Xenopsins are only found in mollusks, other lophotrochozoans, and rotifers (Ramirez *et al*. 2016; Döring *et al*. 2020) and may be expressed in eyes along with r-opsins and c-opsins—depending on the species (Matsuo *et al*. 2019; Döring *et al*. 2020).

The “canonical” G_q_-coupled r-opsins are characteristically used in vision across the invertebrate clades of bilaterians. Previously, duplications of this opsin were identified in the bay scallop, *Argopecten irradians* (Porath-Krause *et al*. 2016), leading to the hypothesis that r-opsin expansion may be a common feature of eye evolution in bivalves. Here, we found only a single r-opsin in the *C. ales* genome vs the four in each of the scallop genomes (Fig. 3b). This result indicates that scallop r-opsin duplication all occurred after the split from Limida. It also demonstrates that recruitment of additional r-opsins is not necessary for eye evolution in Pteriomophia. In fact, with eyeless mytilid species also having multiple r-opsin duplications (Fig. 3b), the role of opsins in ocular vs nonocular processes requires particular attention in Pteriomorphia. The lack of opsin duplication in the *C. ales* genome contributes to the growing evidence for opsin evolution being unrelated to visual complexity in mollusks (De Vivo *et al*. 2023; McElroy *et al*. 2023).

Functional assays of *C. ales* opsins, including in vitro protein expression, as has been performed with scallop opsins (Smedley *et al*. 2022), as well as tissue-specific RNA-seq combined with in situ hybridization and/or immunohistochemistry, will be valuable next steps in determining whether opsins from *C. ales* form photopigments and are expressed in eyes and other light-sensitive tissues.

### First mitogenome from Limida

Mollusks exhibit some of the most variable genomic architecture, molecular functions, and patterns of inheritance for mitochondria in metazoans (reviewed in Ghiselli *et al.* 2021a). Pteriomorphian bivalves are known for dynamic mitochondrial (mt) genome evolution, with Arcidae having repeatedly evolved some of the largest bilaterian mt genomes (Kong *et al*. 2020), and Pectinidae with frequent gene order rearrangements (Malkócs *et al*. 2022) and species with exceptionally large mt genomes (e.g. La Roche *et al*. 1990). Currently, around 300 complete or nearly complete mitochondrial genomes are publicly available on NCBI GenBank, none of which are from Limida. We present the first mt genome assembly for this order of bivalves, which should be a valuable resource for future phylogenetic analyses.

**Fig. 4.**
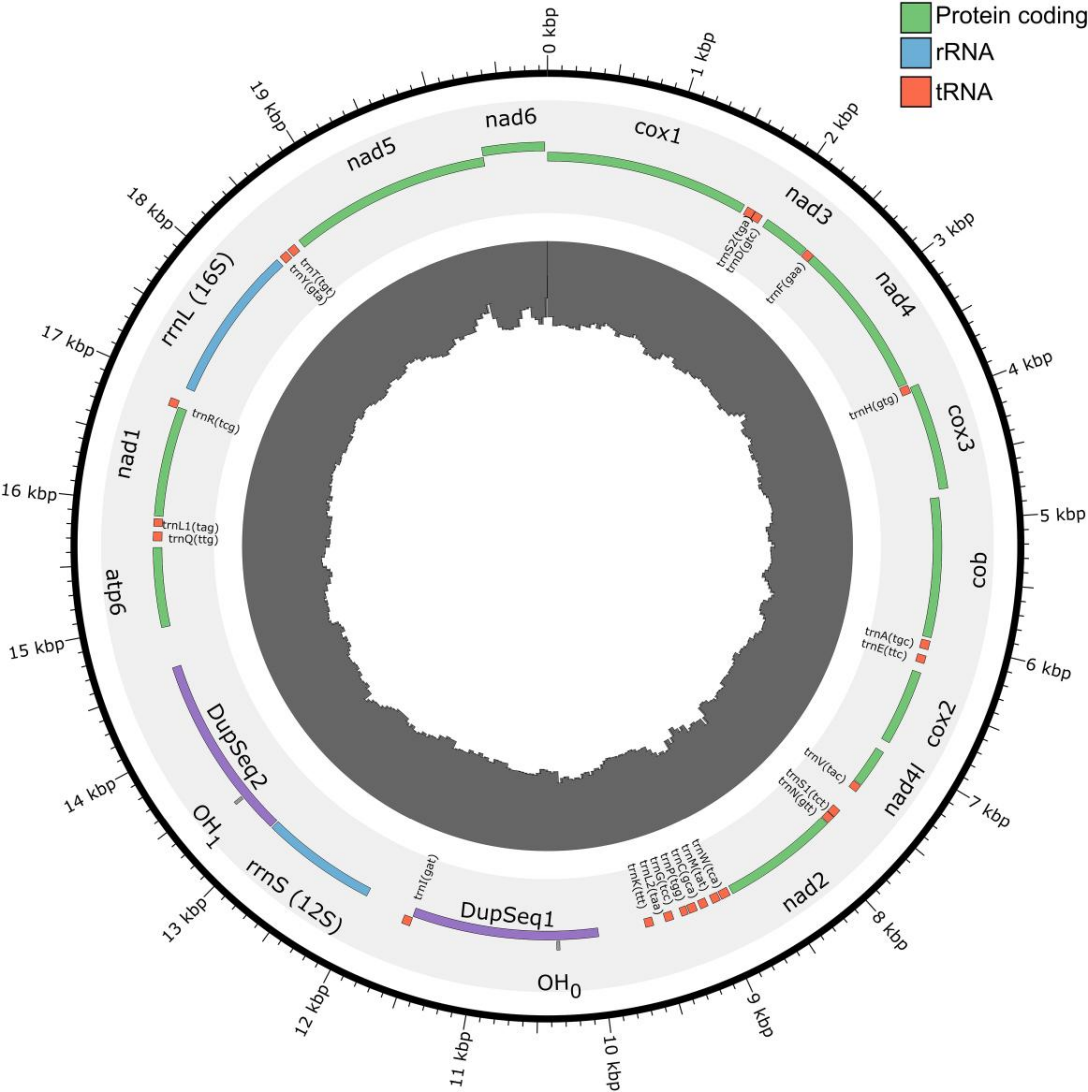
Annotation of *c. ales* mitochondrial genome. Protein-coding, rRNA, and tRNA sequences color coding in legend, duplicated sequence with heavy-strand origin of replication (OH) in purple. Inner histogram of Illumina coverage (2052- 5282X), measured in 100 bp windows step size of 50 bp.

We assembled a 20,859 bp circular contig from the PacBio reads, representing a complete mitochondrial genome sequence. Using a combination of MITOS2, ARWEN, and ORF identification, we annotated 12 complete protein coding genes, the 12S and 16S rRNA genes, and 22 tRNA genes in the *C. ales* mitogenome (Fig. 4). The only typical metazoan protein coding gene not annotated was atp8, which is commonly absent in bivalve mitogenomes (Serb and Lydeard 2003). However, recent analyses support the presence of atp8 sequences in Pectinidae (Malkócs *et al*. 2022) and Mytilidae (Zhao *et al*. 2022).

An interesting feature of this mitogenome is the apparent duplication of the heavy-strand origin of replication (“OH” annotation in MITOS, vs “OL” for light-strand). We found a 1,589 bp sequence duplicated on either side of the 12S rRNA gene that includes an OH annotation from MITOS. The two sequences are 99.8% identical, only differing at three positions. Many (907/4,230) PacBio reads span these repeated sequences (Supplementary Fig. 5). We also observed a 2X relative coverage of one repeat (here, “DupSeq1”) vs the rest of the mt genome when we mapped Illumina reads to an assembly with the second repeat (“DupSeq2”) hard masked (i.e. all nucleotides reported as Ns), which prevented reads from alignment in that region (Supplementary Fig. 6). These results support the accuracy of the mitogenome assembly in having a large duplicated sequence. This duplicated sequence containing OH likely represents the mitogenome “control region,” which regulates replication and transcription (Boore 1999). Duplication of control regions has occurred in a variety of taxa, including birds (Schirtzinger *et al*. 2012), snakes (Jiang *et al*. 2007), and velvet worms (Braband *et al*. 2010), but little is known about the genetic architecture and/or evolutionary pressures underlying and maintaining duplicated control regions. The mitogenome of *C. ales* and limids more broadly may offer insights into the evolution of control regions and further support bivalves as an emerging system for studying mitochondria (Ghiselli *et al.* 2021b).

## Conclusion

In this study, we report a high-quality, chromosome-level assembly for *Ctenoides ales*, the first genome sequenced from the bivalve order Limida. The genome of *C. ales* is noticeably larger than other pteriomorphian bivalves, largely due to a substantial number of transposable elements. We also find that this species has relatively few opsins, compared with other pteriomorphians, indicating that opsin diversification is not guaranteed to accompany the evolution of specialized adult eyes in bivalves. Additionally, we present the complete mitochondrial genome, another first for Limida.

## Supplementary Material

jkae115_Supplementary_Data

## Data Availability

All raw read data have been uploaded to the NCBI SRA database, and the genome assembly has been submitted to NCBI GenBank. Raw reads and assembly are associated with the NCBI BioProject PRJNA1078364. The nuclear and mitochondrial genome assemblies with their annotations, the data underlying species and opsin phylogenies, and scripts used in this study are available on Figshare https://doi.org/10.6084/m9.figshare.25290208. Supplemental material available at G3 online.
