# Supplementary material for: A chromosome-level genome assembly of the disco clam, Ctenoides ales
Source: G3 (Bethesda). 2024 May 28;14(9):jkae115. doi: 10.1093/g3journal/jkae115 (PMC11373642; doi:10.1093/g3journal/jkae115)
Supplement: jkae115_Supplementary_Data [file jkae115_supplementary_data.zip › Supplemental_Figures_G3-2024-405120.pdf]

Figure S1: BUSCO scores for *Ctenoides ales* (bolded) and other bivalves. Purple shading highlights Pteriomorphia.

Figure S2: UpsetPlot comparing shared numbers of orthologs among *C. ales*, Pectinidae, and *Scapharca broughtonii* (Arcidae). The y-axis, or "Intersection size", represents the number of orthogroups shared by a given intersection of species, indicated by the darked dots – connected by lines for intersections > 1. The leftmost column represents orthogroups exclusive to *C. ales*, while the rightmost column represents orthogroups containing genes from all five species included in this figure. Here, most orthogroups include genes from all five species.

Figure S3: Repeat landscapes for **a)** *C. ales*; x-axis: Kimura substitution level of TE copies from representative sequence for distinct family; y-axis: percent of genome. Distribution of TEs reflects past history of activity, where older copies are more diverged from one another (right side of graph) and relatively young copies are less diverged (left side of graph). Apparent ancient and recent bursts of TEs indicated by left and middle peaks. **b)** Representative TE landscapes from other pteriomorphian families: *Pecten maximus* (Pectinidae), *Scapharca broughtonii* (Arcidae), *Crassostrea gigas* (Ostreidae), *Mytilus galloprovincialis* (Mytilidae).

Figure S4: ML (Q.yeast+F+R8 protein substitution model) phylogenetic tree of opsins from 13 pteriomorphian genomes, including *Ctenoides ales*. Generated with IQ-TREE2, ultrafast bootstrap (UFboot) support shown (SH-aLRT and aBayes support included in tree file uploaded to Figshare) Branches color coded according to taxonomic family. *C. ales* opsin names bolded, (*truncated opsin sequences).

Figure S5: Pacbio reads mapped to *C. ales* mitochondria genome assembly. Green lines represent reads that map and span both locations for the duplicated sequences, gray for reads that do not. Annotation of protein-coding genes and the duplicated regions included along the x-axis.

Figure S6: Coverage of duplicated mitochondrial region indicated by Illumina reads. Annotation of *C. ales* mitochondrial genome. Protein-coding, rRNA, tRNA sequences color coding in legend, duplicated sequence with heavy-strand origin of replication (OH) in purple. Inner histogram of Illumina coverage (0-9964), measured in 100bp windows step size of 50bp. Here, DupSeq2 is hard masked (i.e., A/T/C/Gs replaced with Ns) in the assembly to prevent multimapping of reads, demonstrating 2x coverage over DupSeq1 relative to the rest of the mt genome.

Figure S1

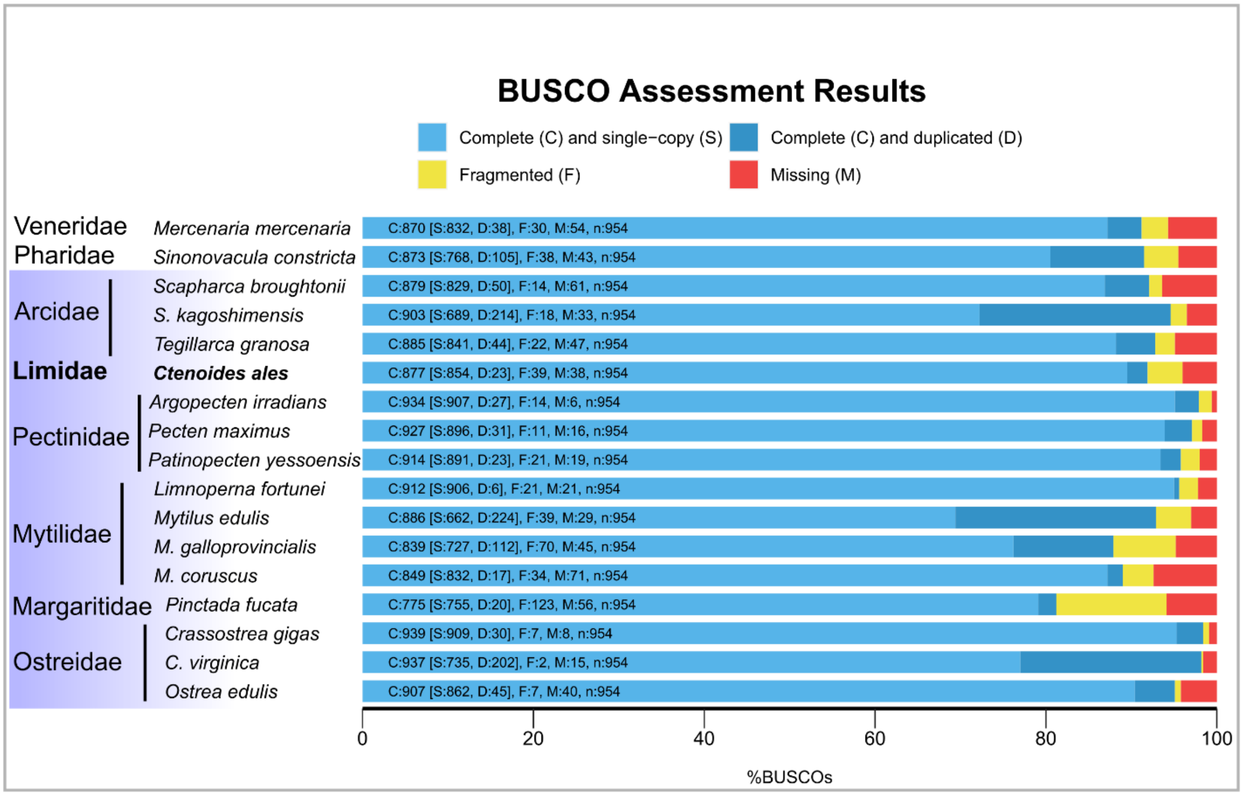
BUSCO Assessment Results
Complete (C) and single−copy (S)
Complete (C) and duplicated (D)
Fragmented (F)
Missing (M)
Veneridae
Mercenaria mercenaria
C:870 [S:832, D:38], F:30, M:54, n:954
Pharidae
Sinonovacula constricta
C:873 [S:768, D:105], F:38, M:43, n:954
Arcidae
Scapharca broughtonii
C:879 [S:829, D:50], F:14, M:61, n:954
S. kagoshimensis
C:903 [S:689, D:214], F:18, M:33, n:954
Tegillarca granosa
C:885 [S:841, D:44], F:22, M:47, n:954
Limidae
Ctenoides ales
C:877 [S:854, D:23], F:39, M:38, n:954
Pectinidae
Argopecten irradians
C:934 [S:907, D:27], F:14, M:6, n:954
Pecten maximus
C:927 [S:896, D:31], F:11, M:16, n:954
Patinopecten yessoensis
C:914 [S:891, D:23], F:21, M:19, n:954
Mytilidae
Limnoperna fortunei
C:912 [S:906, D:6], F:21, M:21, n:954
Mytilus edulis
C:886 [S:662, D:224], F:39, M:29, n:954
M. galloprovincialis
C:839 [S:727, D:112], F:70, M:45, n:954
M. coruscus
C:849 [S:832, D:17], F:34, M:71, n:954
Margaritidae
Pinctada fucata
C:775 [S:755, D:20], F:123, M:56, n:954
Ostreidae
Crassostrea gigas
C:939 [S:909, D:30], F:7, M:8, n:954
C. virginica
C:937 [S:735, D:202], F:2, M:15, n:954
Ostrea edulis
C:907 [S:862, D:45], F:7, M:40, n:954
0
20
40
60
80
100
%BUSCOs

Figure S2

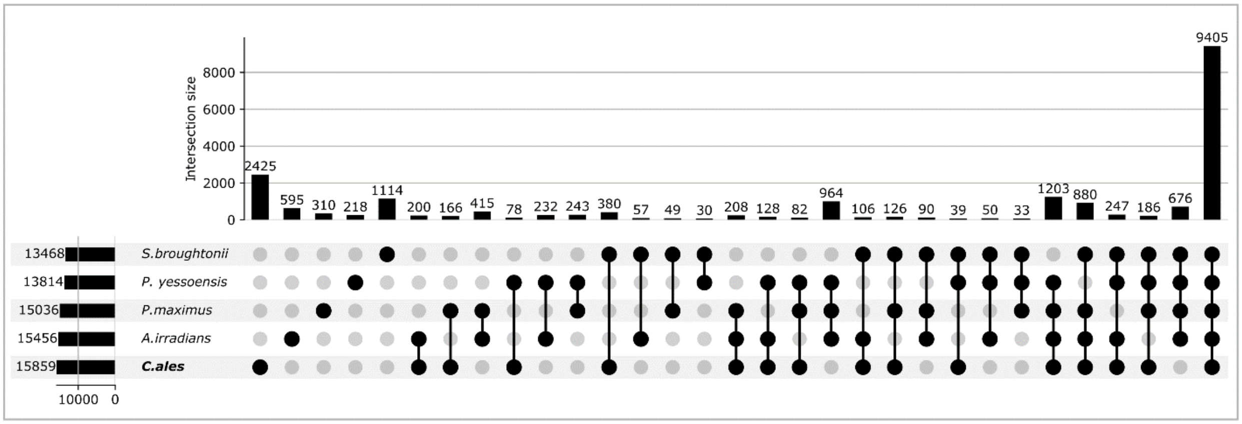
Intersection size
0
2000
4000
6000
8000
2425
595
310
218
1114
200
166
415
78
232
243
380
57
49
30
208
128
82
964
106
126
90
39
50
33
1203
880
247
186
676
9405
13468
13814
15036
15456
15859
S.broughtonii
P. yessoensis
P.maximus
A.irradians
C.ales
10000
0

Figure S3

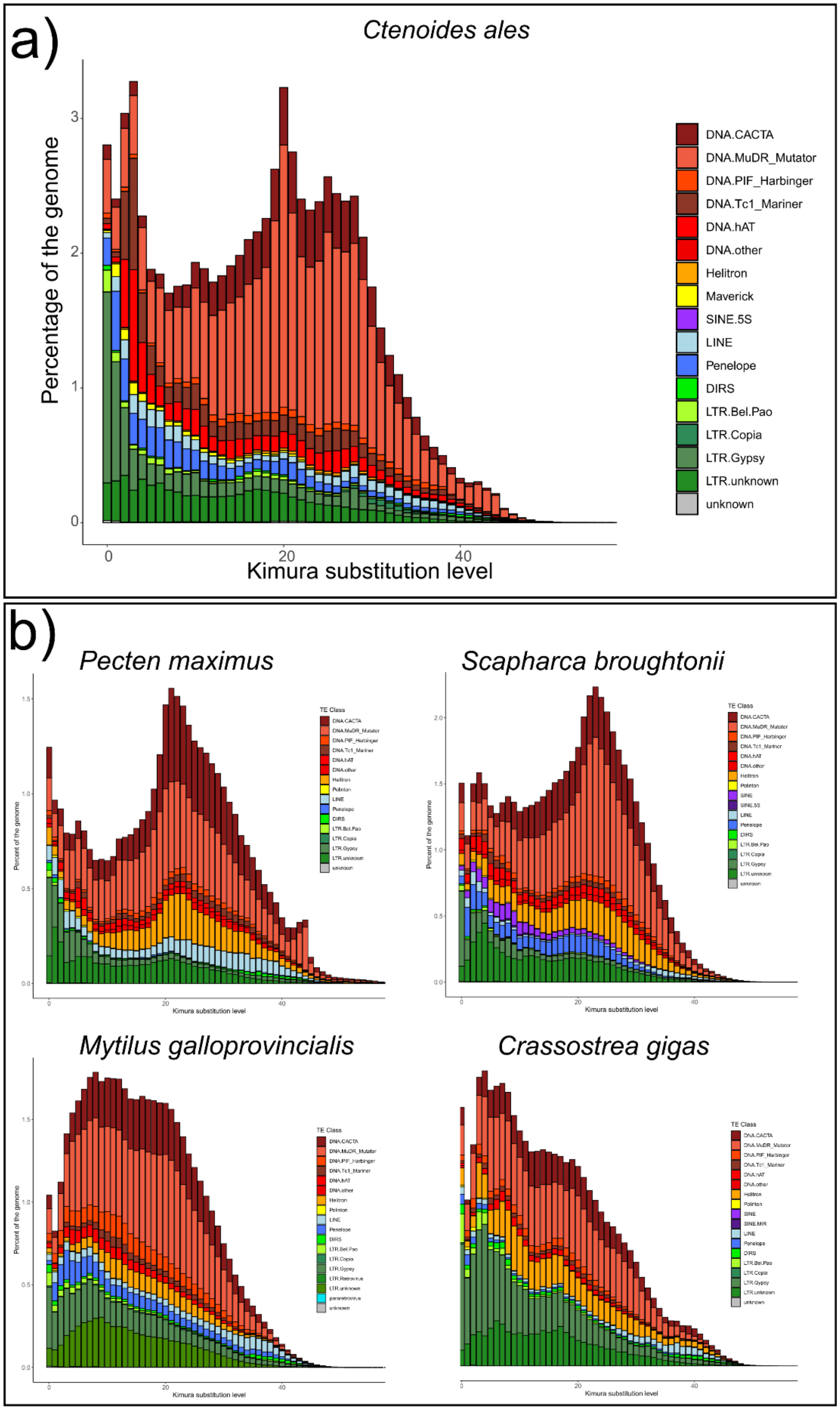
a)
Ctenoides ales
Percentage of the genome
Kimura substitution level
0
1
2
3
0
20
40
DNA.CACTA
DNA.MuDR_Mutator
DNA.PIF_Harbinger
DNA.Tc1_Mariner
DNA.hAT
DNA.other
Helitron
Maverick
SINE.5S
LINE
Penelope
DIRS
LTR.Bel.Pao
LTR.Copia
LTR.Gypsy
LTR.unknown
unknown
b)
Pecten maximus
Percent of the genome
Kimura substitution level
TE Class
DNA.CACTA
DNA.MuDR_Mutator
DNA.PIF_Harbinger
DNA.Tc1_Mariner
DNA.hAT
DNA.other
Helitron
Polinton
LINE
Penelope
DIRS
LTR.Bel.Pao
LTR.Copia
LTR.Gypsy
LTR.unknown
unknown
Scapharca broughtonii
Percent of the genome
Kimura substitution level
TE Class
DNA.CACTA
DNA.MuDR_Mutator
DNA.PIF_Harbinger
DNA.Tc1_Mariner
DNA.hAT
DNA.other
Helitron
Polinton
SINE
SINE.5S
LINE
Penelope
DIRS
LTR.Bel.Pao
LTR.Copia
LTR.Gypsy
LTR.unknown
unknown
Mytilus galloprovincialis
Percent of the genome
Kimura substitution level
TE Class
DNA.CACTA
DNA.MuDR_Mutator
DNA.PIF_Harbinger
DNA.Tc1_Mariner
DNA.hAT
DNA.other
Helitron
Polinton
LINE
Penelope
DIRS
LTR.Bel.Pao
LTR.Copia
LTR.Gypsy
LTR.Retrovirus
LTR.unknown
pararetrovirus
unknown
Crassostrea gigas
Kimura substitution level
TE Class
DNA.CACTA
DNA.MuDR_Mutator
DNA.PIF_Harbinger
DNA.Tc1_Mariner
DNA.hAT
DNA.other
Helitron
Polinton
SINE
SINE.MIR
LINE
Penelope
DIRS
LTR.Bel.Pao
LTR.Copia
LTR.Gypsy
LTR.unknown
unknown

Figure S4

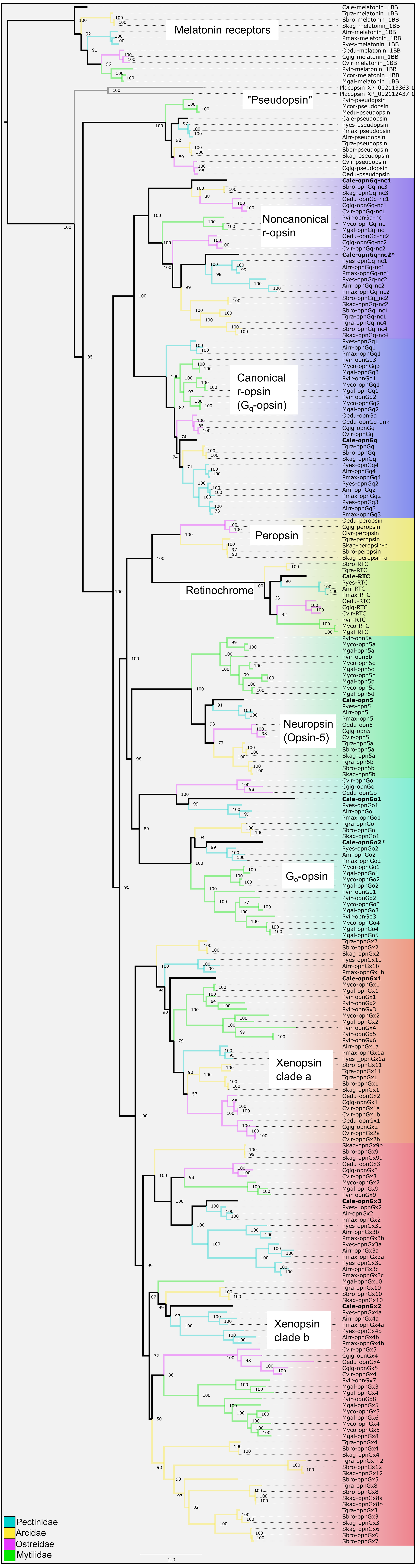

Melatonin receptors
"Pseudopsin"
Noncanonical r-opsin
Canonical r-opsin (G_q-opsin)
Peropsin
Retinochrome
Neuropsin (Opsin-5)
G_o-opsin
Xenopsin clade a
Xenopsin clade b
Pectinidae
Arcidae
Ostreidae
Mytilidae
2.0

Figure S5

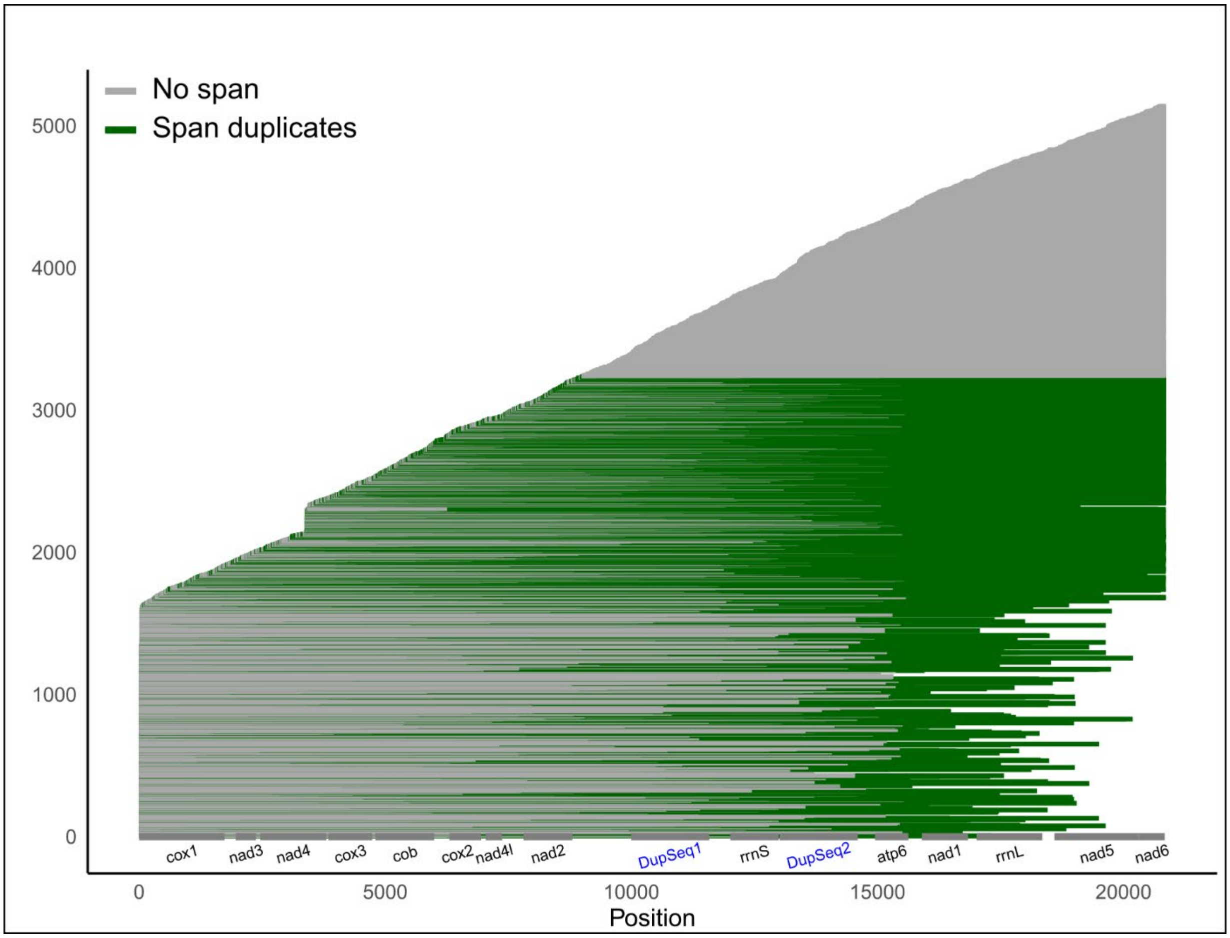

No span
Span duplicates
5000
4000
3000
2000
1000
0
cox1
nad3
nad4
cox3
cob
cox2
nad4l
nad2
DupSeq1
rrnS
DupSeq2
atp6
nad1
rrnL
nad5
nad6
0
5000
10000
15000
20000
Position

Figure S6

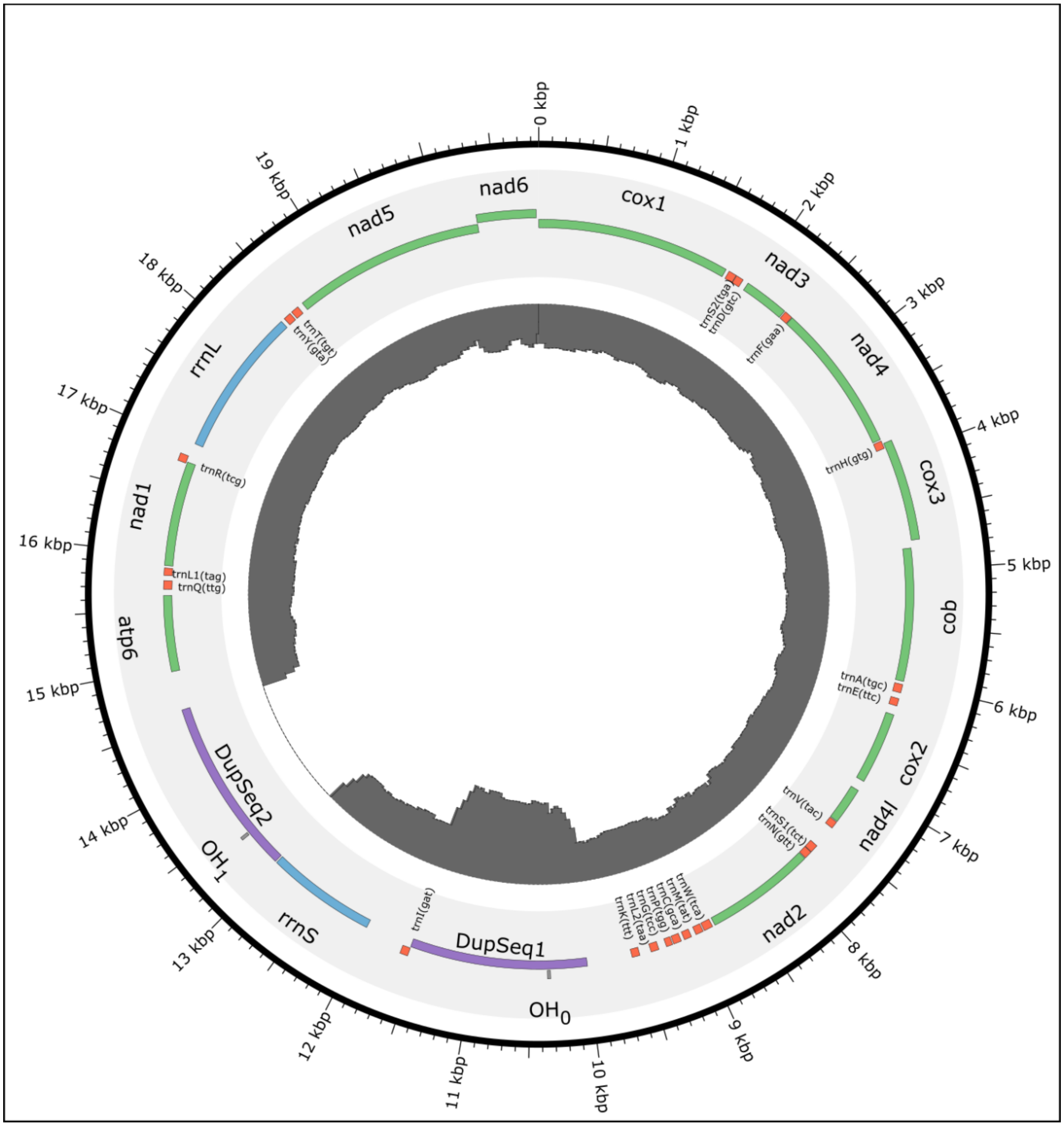

0 kbp
1 kbp
2 kbp
3 kbp
4 kbp
5 kbp
6 kbp
7 kbp
8 kbp
9 kbp
10 kbp
11 kbp
12 kbp
13 kbp
14 kbp
15 kbp
16 kbp
17 kbp
18 kbp
19 kbp
nad6
cox1
nad5
nad3
trnS2(tga)
trnD(gtc)
trnF(gaa)
nad4
trnT(tgt)
trnY(gta)
rrnL
trnH(gtg)
cox3
trnR(tcg)
nad1
cob
trnL1(tag)
trnQ(ttg)
atp6
trnA(tgc)
trnE(ttc)
cox2
DupSeq2
trnV(tac)
nad4l
trnS1(tct)
trnN(gtt)
OH1
rrnS
trnI(gat)
trnW(tca)
trnM(tat)
trnC(gca)
trnP(tgg)
trnG(tcc)
trnL2(taa)
trnK(ttt)
nad2
DupSeq1
OH0
